# Construction of a near infrared fluorescence system for imaging of biological tissues

**DOI:** 10.1038/s41598-024-51583-w

**Published:** 2024-01-18

**Authors:** Xu Zhao, Shilin Li, Yue Song, Lianhui Fan

**Affiliations:** 1 Department of Urology, General Hospital of Northern Theater Command, Shenhe District, No. 83, Wenhua Rd, Shenyang, 110000 China; 2 Department of Graduate School, China Medical University, Shenyang, China

**Keywords:** Anatomy, Gastroenterology, Medical research, Urology

## Abstract

Surgical procedures often rely on unaided visual observation or endoscopic assistance, which may pose challenges in cases involving intricate anatomical relationships. Real-time imaging technologies capable of intraoperative visualization of target organs have the potential to enhance the precision of surgical procedures by facilitating accurate identification, separation, and protection of vital tissues or organs. Despite these advantages, the widespread adoption of such technologies has been hindered by factors such as the prohibitive cost of equipment. This study aims to optimize and develop a device based on Indocyanine Green (ICG) for fluorescence imaging. The objective is to monitor changes in the average fluorescence intensity of ICG in the bladder, offering valuable guidance for surgeries involving the bladder. 1. Male rabbits were administered 0.01 mg/ml ICG via the renal pelvis and ear vein to obtain fluorescence images of the ureter, bladder, and small intestine. 2. After ligating the bilateral ureters of male rabbits, a retrograde bladder perfusion of 5 ml 0.01 mg/ml ICG was conducted to capture fluorescence images of the bladder over time. The average fluorescence intensity was computed using Image Pro Plus 6.0, and the corresponding curve was generated using Prism 8.0. Using a similar methodology, the average fluorescence intensity of male rabbits without ureteral ligation was measured and plotted over time. 1. The developed device facilitated imaging of the ureter, bladder, and small intestine. 2. The bladder's average fluorescence intensity exhibited changes over time in response to urine production and ureteral ligation, contrasting with observations without ureteral ligation. We have successfully constructed and optimized a modular fluorescence imaging system for organs and tissues. This system proves effective in imaging experiments involving hollow organs in animals and offers valuable insights for relevant surgical procedures.

## Introduction

Under the current technical constraints, surgeons rely on unaided vision or endoscopic assistance for surgical procedures. In cases involving intricate anatomical relationships, accurate identification, separation, and protection of target tissues or organs can be challenging using visual methods alone. Tumor resection poses difficulties in sensitively detecting tumor sentinel lymph nodes, accurately locating tumor blood vessels, and confirming real-time completion of tumor removal. Trauma patients face challenges in observing blood perfusion in injured tissues or organs in real-time and dynamically assessing tissue necrosis objectively. In addressing these challenges, intraoperative real-time imaging of tissues or blood vessels holds distinct technical advantages.

Indocyanine Green (ICG) is an anionic water-soluble tricarbonyl cyanine molecule belonging to the near-infrared spectrum fluorescent dye category. Developed by Kodak Laboratories in 1955, ICG has received recognition from the US Food and Drug Administration (FDA)^1^. Following intravenous administration, ICG selectively binds with plasma proteins in the vascular lumen and rapidly distributes throughout the body's blood vessels. It is taken up by liver cells and excreted into bile in a free form, ultimately being eliminated from the body via feces^2^. Similar to Egloff-Juras's research, our findings indicate that when ICG is excited by short-wavelength near-infrared light (e.g., 780 nm), it emits near-infrared fluorescence of a relatively long wavelength (e.g., 820 nm)^3^. Leveraging the imaging method of Indocyanine Green Video Angiography (ICGVA), real-time imaging of biological tissues and organs becomes achievable. ICGVA technology finds applications in trauma diagnosis and treatment related to natural or man-made disasters, including identifying ischemic and necrotic tissues^4,5^, assessing blood perfusion in frostbitten tissues, and evaluating organ trauma severity^6,7^. Additionally, ICGVA aids in protecting hollow organs such as the ureter^8^ and has been employed in various cancer-related surgeries, including liver cancer^9^, renal cancer^10^ and cervical cancer^11^.

**Figure 1:**
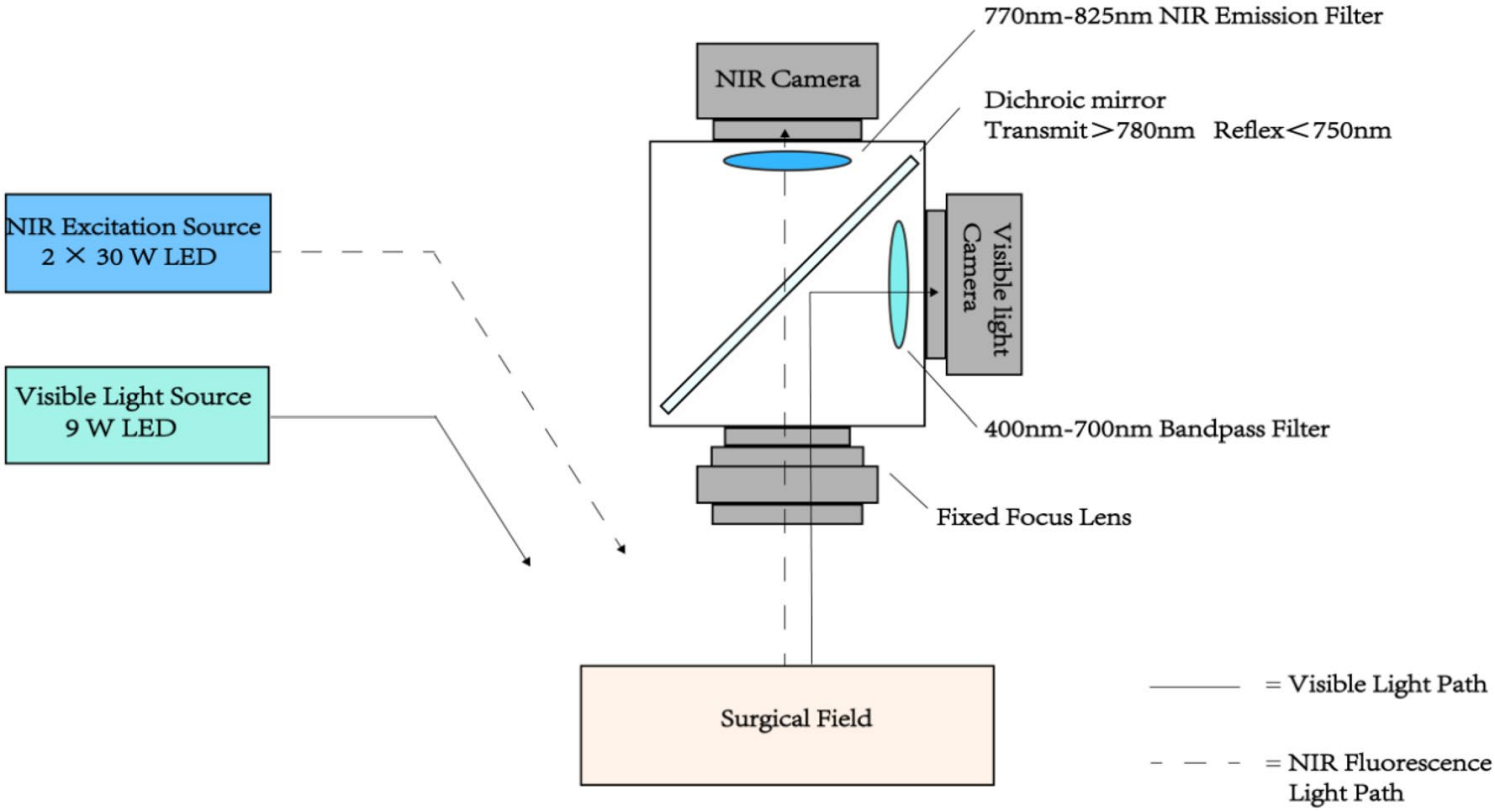
Optical Path Schematic Diagram: Visible light initially interacts with the surface of the test sample, undergoes reflection off the surface, propagates along the visible light path, is reflected by a dichroic mirror, and ultimately captured by a visible light camera. Simultaneously, near-infrared light reaches the surface of the test sample. Upon fluorescence irradiation, ICG within the tissue undergoes a spectral transition, emitting longer-wavelength near-infrared light. This beam propagates along the near-infrared light path, passes through the dichroic mirror, and is eventually captured by a near-infrared camera.

However, the widespread adoption of intraoperative real-time development technology is hindered by factors such as high equipment costs, complex supporting devices, and poor portability. Grand et al. attempted to address these challenges by designing a near-infrared fluorescence imaging device using ICG as the imaging material, applied successfully in large animals such as pigs for vascular imaging^12^. Nonetheless, their device has limitations, including poor universality and portability. In our redesign of the entire system (see Fig. 1), we have optimized platform mobility, reduced costs, enhanced positioning accuracy, and improved the light source and optical path system. We validated the feasibility of this redesigned device using rabbits, conducting imaging of organs such as the intestine, bladder, and ureter. Notably, changes in ICG fluorescence intensity in the bladder offer valuable assistance in bladder-related surgical operations.

## Materials and methods

Table 1 lists the relevant materials involved in this equipment.

**Table 1:** Imaging element parameters.

Focusing platform	
Size Weight Power supply Working distance	35 cm L × 30 cm W × 100 cm H 15 kg 220 V AC, rated voltage 250 V, maximum current 10 A 5–20 cm (Distance between near-infrared excitation light source and experimental platform) 55.8 ± 1.8 cm (Distance between the fixed focus lens and the experimental platform)
Optical path system	
Visible light source power	9 W LED
Central wavelength of visible light source	450 ± 10 nm
NIR excited light source power	2 × 30 W LED
Central wavelength of NIR excited light	750 ± 10 nm
V-interface line scanning fixed focus lens	ELS-100/5.6 V Resolution: 40 IP/mm Focal length: 100 mm Magnification: 0.17 ×
Aluminum dark box	Weight: 106.5 g 60 mm L × 52 mm W × 52 mm H
Dichroic mirror	Transmission > 780 nm Reflection < 750 nm
Filter	770—1000 nm ± 10 nm (near-infrared light) 400—700 nm ± 10 nm (visible light)
Camera	BIGEYE10000KPA Sensor: 10.3 M/IMX294 Pixel: 4.63 μm × 4.63 μm Resolution: 1360 × 720 Exposure time: 0.1 ms ~ 15 s

General Hospital of Northern Theater Command approved the experiment.and the experiment was performed in accordance with relevant named guidelines and regulations.

The authors complied with the ARRIVE guidelines.

### Three-dimensional adjustable focus vision platform

**Figure 2:**
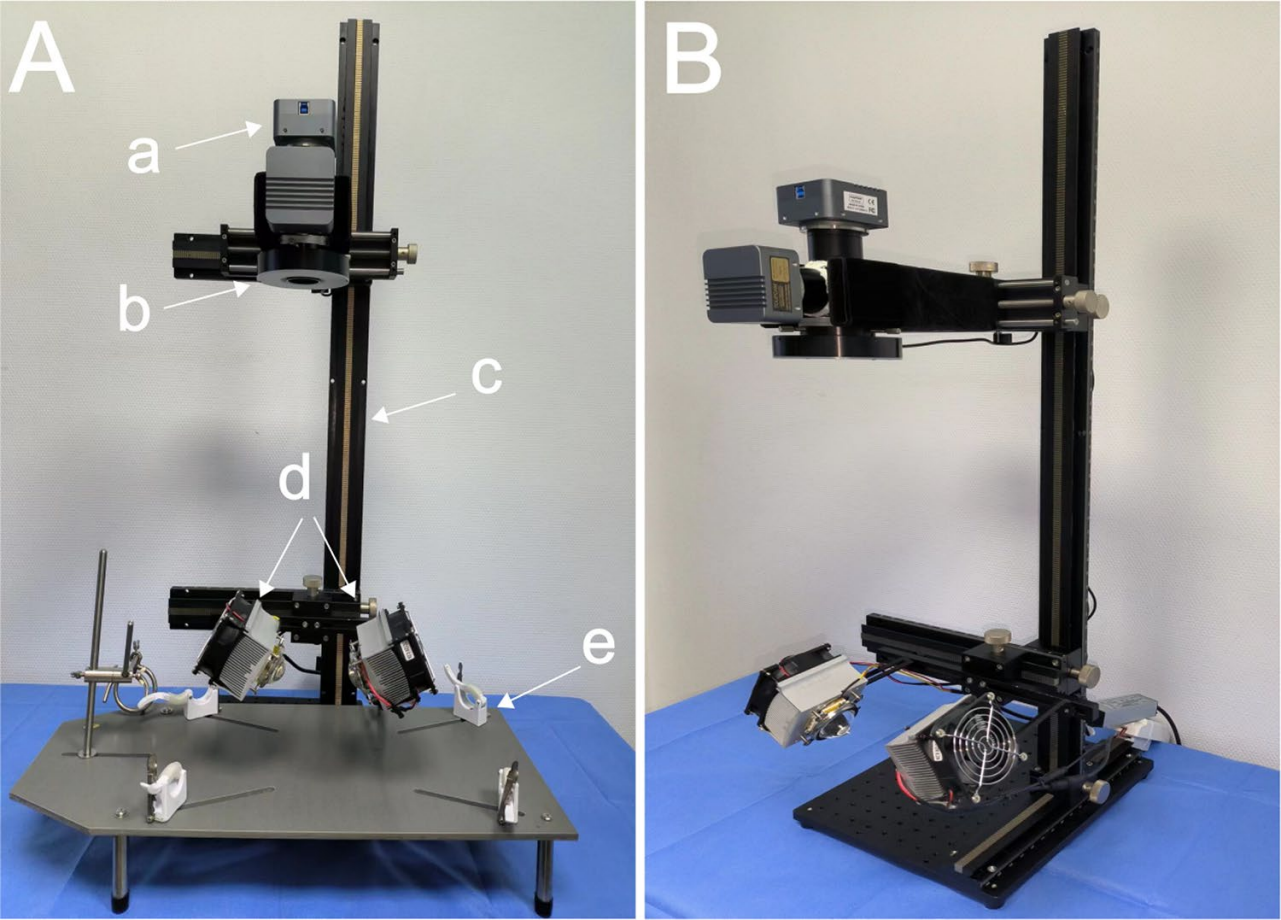
The comprehensive structure of the fluorescence imaging system is outlined as follows: (**A**) Front View of the 3D Adjustable Focus Vision Platform: a. Optical Path System: Receives visible light and fluorescent light, generating corresponding visible light and fluorescent images. b. 9 W Visible Light Source. c. Customized Movable Platform. d. Two Groups of 30 W Power LED Near-Infrared Excitation Light Sources and Supporting Heat Dissipation Device. e. Surgical Field. (**B**) Side View of the 3D Adjustable Focus Vision Platform.

The adjustable focus vision platform comprises two layers of removable platforms (customized version, Bianteng Technology Co., Ltd., Jiangsu, China) as illustrated in Fig. 2A. The upper movable platform is primarily designed to secure imaging modules, encompassing cameras, lenses, and the dichroic mirror, along with the visible light source. Meanwhile, the lower movable platform is dedicated to stabilizing the near-infrared light sources. The camera clip (Fig. 2Aa) on the upper platform is equipped to move and finely adjust within the horizontal plane (along the X and Y axes) and vertically (along the Z axis, as depicted in Fig. 2Ac). Similarly, the near-infrared light source on the lower platform is capable of horizontal and vertical adjustments. The entire visual focusing platform can function independently or be affixed to a removable platform. Each component is disassemblable, facilitating customization and combination according to surgical requirements.

### Light source system

**Figure 3:**
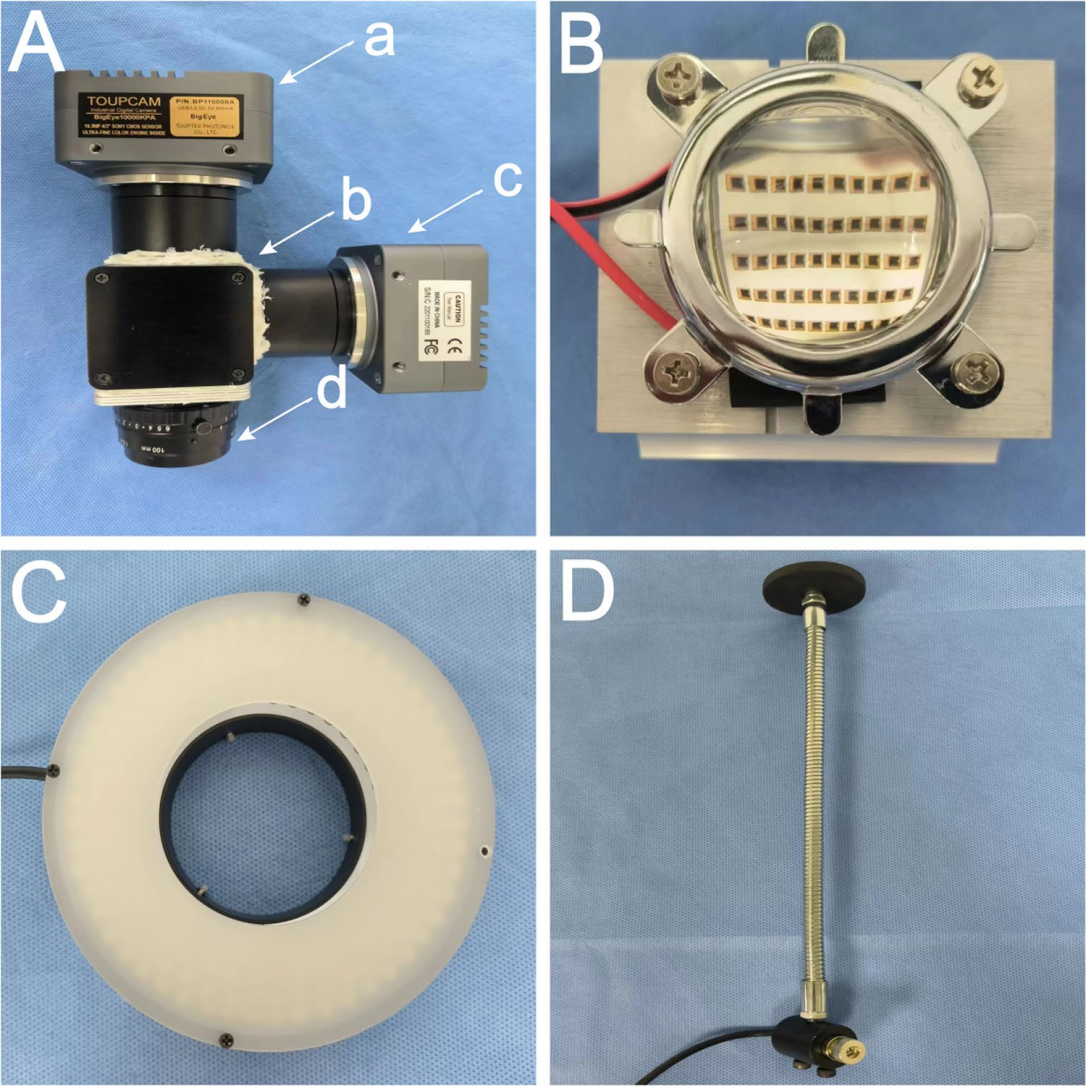
Components of the Fluorescence Imaging System: (**A**) Optical Path System: a. NIR Camera, connected with 770-825 nm band-pass filter. b. Aluminum Black Box, housing a long wave pass dichroic mirror (5 × 10–2 6 cm, 400—750 nm HR, 780—900 nm HT). c. Visible Light Camera, connected with 400–700 nm band-pass filter. d. V-interface Line Scanning Fixed Focus Lens, receiving visible light and fluorescent light. (**B**) NIR Excitation Source: 30 W LED, equipped with a 60° lens and a central wavelength of 753 nm. (**C**) Visible Light Source: 9 W Circular LED, with an inner diameter of 53 mm, five rows of lamp beads, and a central wavelength of 452 nm. (**D**) Cross Laser Locator.

The near-infrared excitation light source is composed of a square array of near-infrared LED beads, while the visible light source comprises a circular array of visible LED beads. To meet diverse experimental requirements, the near-infrared excitation light source allows for flexible adjustments in the number of modules, placement position, total output energy, light wavelength band, front surface lens scattering angle, and other indicators. In this study, two sets of 30 W power LED near-infrared excitation light sources (equipped with a 60° lens and a central wavelength of 753 nm) and accompanying heat dissipation devices (customized version, Kiwi Optoelectronics Co., Ltd., Shenzhen, China) are positioned above the operating platform. This arrangement ensures high power intensity and comprehensive light coverage on the surgical plane (Fig. 3B). The visible light source comprises a 9 W circular LED light source (model A53108, inner diameter 53 mm, five rows of lamp beads, central wavelength 452 nm, Guangzhou Limin Resources Co., Ltd., Guangdong, China) situated below the lens (Fig. 3C). The light intensity of the source is adjustable to guarantee optimal illuminance on the test sample's surface.

During the experiment, both the visible light source and the near-infrared light sources are activated simultaneously, allowing direct observation of the tissue by the naked eye. Subsequently, images captured by two cameras display real-time tissue images under visible light and near-infrared light, respectively.

### Optical path system

The imaging module is situated on the upper movable platform of the experimental bench, capable of capturing visible light and near-infrared fluorescence to generate corresponding images. The module is constructed using an aluminum black box (customized version, Fengyun shell, Ningbo, Zhejiang, China) as the central functional component connected in multiple directions (Fig. 3Ab). The inner surface of the dark box is black anodized to ensure light-tightness and non-reflectivity, minimizing external interference. The dark box's dimensions are 60 mm in length, 52 mm in width, and 52 mm in height. Circular holes with diameters of 32 mm, 40 mm, and 40 mm are provided on the top, bottom, and side faces, respectively. Within the dark box, a long-pass dichroic mirror (5 × 6 cm, 400–750 nm HR, 780–900 nm HT), a 770–825 nm band-pass filter (model 102, diameter 12.7 mm, United Light Technology Co., Ltd., Beijing, China), and a near-infrared CMOS camera (BIGEYE10000KPA, Toup Tek Photonics, Zhejiang, China) are connected on the top face. Additionally, a 400–700 nm band-pass filter (United Light Technology Co., Ltd., Beijing, China) and a visible light camera (BIGEYE10000KPA, Toup Tek Photonics, Zhejiang, China) are connected on the side face, while a V-interface line-scanning focusing lens (ELS-100/5.6 V, Haolan Optoelectronics Co., Ltd., Fujian, China) is connected on the bottom face (Fig. 3Ad).

During the experiment, visible light initially strikes the object's surface, reflects off it, propagates along the visible light path, and is captured by the visible light camera. Simultaneously, near-infrared light reaches the object's surface, and after fluorescence irradiation caused by ICG in the tissue, a spectral transition occurs, emitting longer-wavelength near-infrared light. This beam travels along the near-infrared light path and is eventually captured by the near-infrared camera.

### Laser positioning system

To ensure precise machine vision localization of the center point within the region of interest (ROI), a removable cross laser locator (red light 650 nm, Xinkunyang Technology Co., Ltd., Shenzhen, China) was installed at the same horizontal plane as the dichroic mirror (Fig. 3D). Prior to surgery, the cross-center point of the laser can be adjusted to align with the imaging center point under visible light, and the laser locator can then be securely fixed. During the surgical procedure, as the camera moves, the laser cross's location point within the surgical area serves as the imaging center point. This system significantly enhances the efficiency and accuracy of ROI positioning during surgery.

### Computer processing system

We have developed a LabVIEW 4.11.18012 program as the control software (Fig. 5). This program seamlessly orchestrates the operations of both the near-infrared camera and the visible camera simultaneously. Utilizing the software, we achieve technical fusion imaging of target tissues or blood vessels by digitally combining visible and near-infrared images. Initially, the LabVIEW software is configured to capture fluorescent grayscale images, wherein the program eliminates the black background, retaining only the fluorescent components. Subsequently, the fluorescent section is pseudo-colored and fused with the visible light color image. The result is a fusion image that overlays the pseudo-colored fluorescent image onto the color visible light image. The software provides flexibility to adjust various imaging parameters, including sampling frequency, white balance, brightness, contrast, gamma value, rotation angle, translation or rotation replacement value, etc., ensuring the capture of precise and clear images. Throughout the imaging process, the device achieves a frame rate of 2.8 Hz, and the spatial resolution (image size in pixels) reaches 4096 × 2160 DPI.

### Intraoperative fluorescence imaging

1. We selected 10 male rabbits (3.0 ± 0.2 kg), numbered 1–10, and prepared ICG solutions with concentrations of 0.001, 0.0025, 0.005, 0.01, 0.02, 0.05, 0.10, 0.25, 0.5, and 1.0 mg/ml, respectively. Following the ligation of the ureters on both sides of male rabbits numbered 1–8, we retrogradely perfused 5 ml (sufficient to fill the bladder) of each of the 10 concentrations of ICG solution into the bladder to obtain initial bladder fluorescence images. Image Pro Plus 6.0 was utilized to measure the average fluorescence intensity of the bladder at different concentrations. Our findings indicated a gradual increase in the average fluorescence intensity with 0.001 mg/ml ICG. Compared with lower concentration ICG, the average fluorescence intensity of 0.01 mg/ml ICG was higher. Furthermore, in comparison with higher concentration ICG, there was no significant change in the average fluorescence intensity of 0.01 mg/ml ICG, allowing for the achievement of complete bladder fluorescence imaging. Consequently, we selected 0.01 mg/ml ICG for the experiment.
2. We selected typical hollow organs, such as the ureter, bladder, and small intestine, for imaging. Eight male rabbits (2.0 ± 0.5 kg) were used and randomly divided into two groups: Group 1 for urinary system fluorescence imaging (n = 4) and Group 2 for small intestine fluorescence imaging (n = 4). Anesthesia was induced with a 0.3 ml/kg hydrochloric acid methylthiazide intramuscular injection. In Group 2, 2 mL of 0.01 mg/mL ICG was injected into the bilateral renal pelvises of rabbits, and images of the ureter and bladder were captured. In Group 1, 2 mL of ICG (0.01 mg/mL) was injected into the ear vein of rabbits, and images of the small intestine were captured.
3. We focused on the bladder for further imaging experiments. Six male rabbits (2.0 ± 0.5 kg) were randomly divided into two groups. Each rabbit underwent a 24-h water fast. During the experiment, the NIR excitation light source was positioned 7 cm away from the rabbits, and the fixed focus lens was set 56 cm away from the experimental platform. In Group 1 (n = 3), retrograde infusion of 5 ml of 0.01 mg/ml ICG into the bladder (keeping the bladder just filled) was performed, followed by fluorescence imaging to observe the fluorescence changes in the bladder from the initial to macroscopic stages, providing a comprehensive visual field for surgery. The average fluorescence intensity at the corresponding time was calculated. In Group 2 (n = 3), retrograde infusion of 5 ml of 0.01 mg/ml ICG into the bladder occurred after bilateral ureteral ligation. Fluorescence imaging was conducted to obtain fluorescence images at each point, and the corresponding average fluorescence intensity was calculated. Finally, the average fluorescence intensity of the two groups of fluorescence images was compared.

## Results

### Optimization of elements and reagents of imaging equipment

#### The adjustable focal length experimental platform

The utilization of this adjustable focal length experimental platform offers a distinct advantage wherein the experimental animal remains stationary, while the light source and camera can be moved and fine-tuned in multiple directions within three-dimensional space. This design enables the maintenance of linkage between the light source and camera, thereby enhancing the control capability of the platform and the precision imaging ability of machine vision (Fig. 2B).

#### Light source

To mitigate non-uniformity and shadows in the surgical area, a shadowless light source system is implemented using two sets of LED near-infrared excitation light sources and one set of LED visible light sources (Fig. 2Ab,d). In contrast to halogen light sources^12^, the customized LED light source offers advantages such as a narrow spectrum, low energy consumption, high brightness, minimal heat generation, extended service life, and easy control.

#### Heat dissipation system

The air cooling system incorporates a fan (82*82*25 mm) tailored to the size of the LED excitation light source, maintaining a stable fan speed at 900 RPM. The air-cooled radiator and LED light source initiate synchronously, effectively minimizing the load resulting from the excitation light source's heating. This synchronized operation ensures the stability and safety of the light source(Fig. 2Ad).

#### Optical path system

The C-interface lens has a back intercept that is too short (17.5 mm) to accommodate a dichroic mirror between the camera CMOS element and the lens. On the contrary, the V-interface line scanning fixed focus lens features relatively larger diameters and longer back intercepts. This allows for the insertion of a dichroic mirror between the camera and the lens, enabling the camera to capture normal images effectively. This enhancement significantly improves lens compatibility and applicability(Fig. 3Ad).

In the working distance range of 5–20 cm, ICG exhibits fluorescence transition from the unexcited state to the excited state within a time frame of 1.18 s. Notably, the sensitivity remains consistent across different working distances.

#### Camera sensitivity range and software adaptability

**Figure 4:**
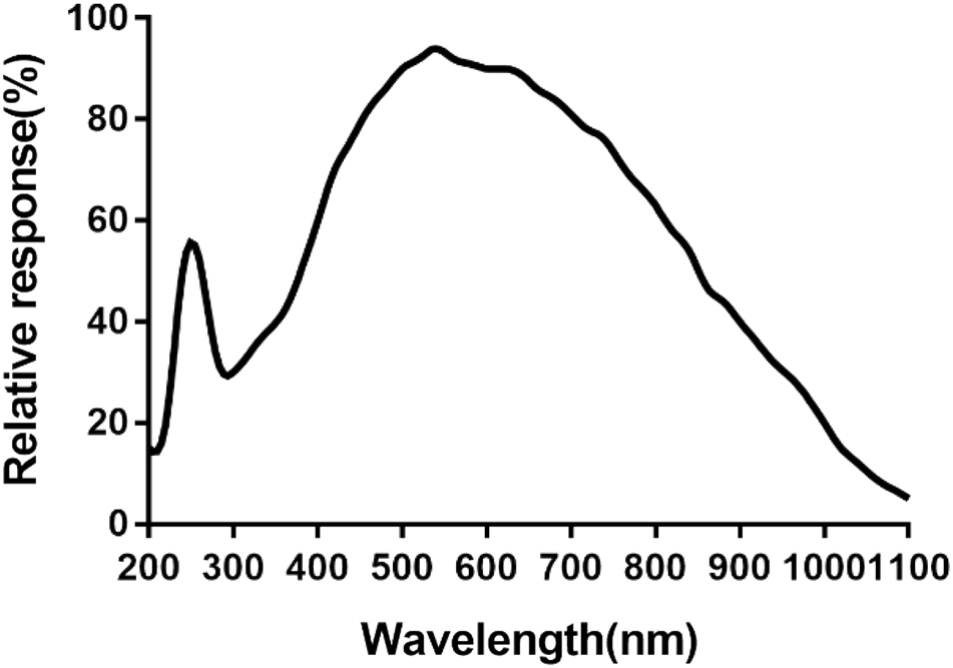
Spectral Response Curve of Camera: The graph depicts the relationship between wavelength (abscissa) and relative photosensitive effect (ordinate). The spectral response curve of the BIGEYE10000KPA camera spans from 200 to 1100 nm. This sensitivity range effectively covers both the visible spectrum range of 380—780 nm and the near-infrared spectrum range of 780–1100 nm.

The BIGEYE10000KPA camera exhibits a spectral response range from 200 to 1100 nm, as illustrated in Fig. 4. Its sensitivity range comprehensively covers both the visible spectrum (380—780 nm) and the near-infrared spectrum (780–1100 nm). This camera is well-suited for observing both visible light and near-infrared fluorescence. It integrates a 12-bit Ultra-Fine hardware image signal processor video streaming engine (Ultra-Fine HISPVP) and utilizes USB 3.0 data transmission technology for efficient data transfer. Notably, no significant delay is observed in video transmission. The advantages of this camera include cost savings, parameter consistency across machines, and uniform image quality between visible light imaging and fluorescent imaging.

#### Standardization of mechanical elements and reagents

The imaging system we have developed allows for flexible adjustments of relative reagent types and parameters for various equipment components, eliminating the need for fixed standards. All parameters can be tailored to specific experimental or clinical requirements. For instance, standardized industrial products can be chosen for the original parts of imaging equipment, such as C-interface cameras and various band-pass filters. The accessories possess characteristics such as low cost, good versatility, and strong replaceability. Experimental consumables, including ICG and other reagents, can be easily substituted with a series of derivatives based on actual needs. Consequently, corresponding bandpass filters are employed to complete the imaging process. This adaptability ensures the system's versatility and applicability in diverse experimental and clinical scenarios.

#### The portability of the equipment

The imaging equipment components boast advantages such as compact size, lightweight design, and detachability. They can be easily installed on a customized removable cart based on specific requirements. The external accessory consists of a laptop (compatible with Windows or Mac) with LabVIEW software installed. The entire set of equipment is straightforward and removable. This simplicity and portability make it particularly valuable in applications involving extreme environments, such as emergency rescue under challenging conditions. In such scenarios, this equipment set can leverage its unique advantages by providing crucial imaging information for decision-making in injury control surgery.

#### Fluorescence imaging of bladder and other hollow organs

**Figure 5:**
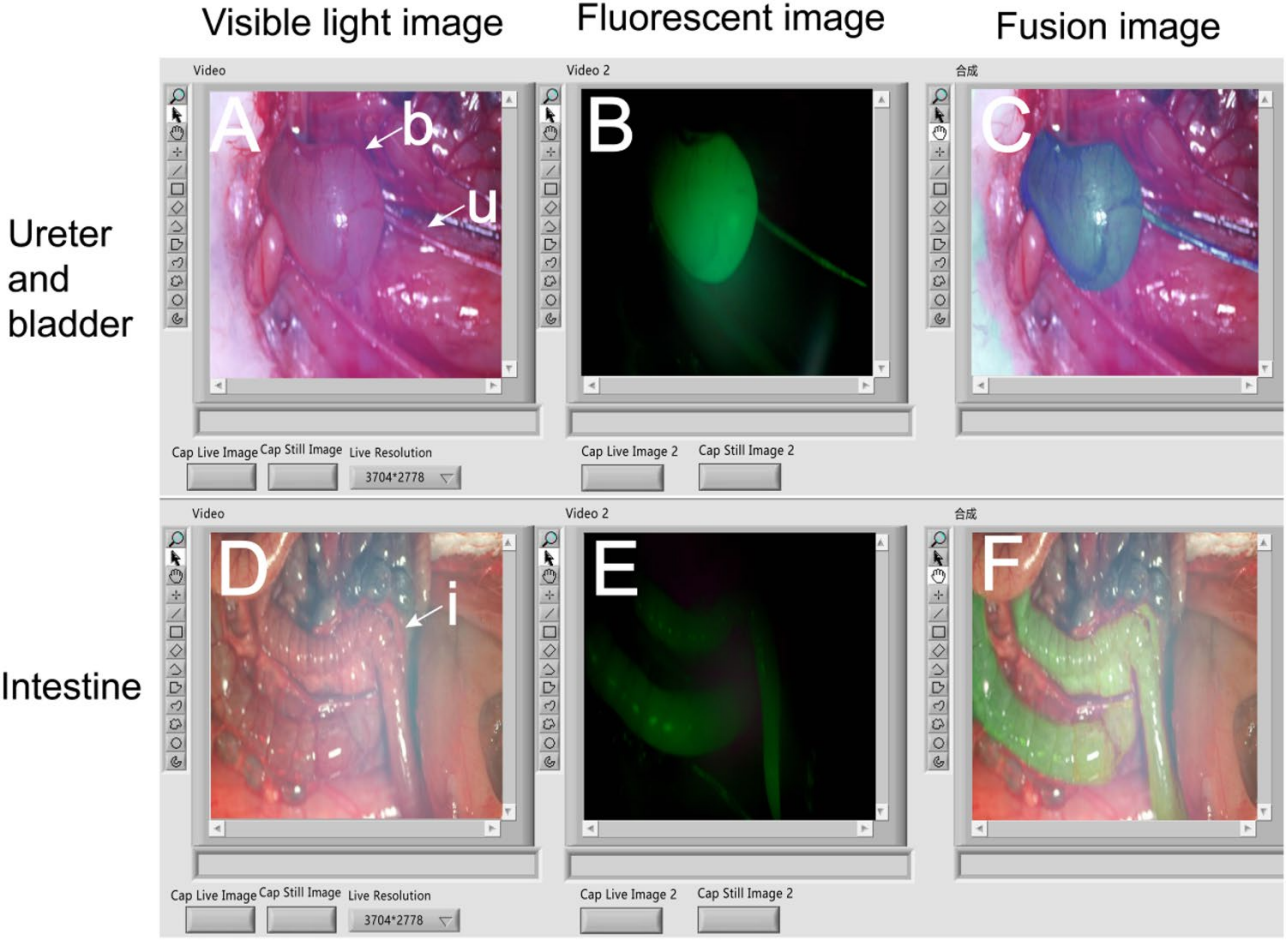
LabVIEW Software Image Display: (**A**–**C**) Visible light image, near-infrared fluorescence image, and fusion image of the ureter and bladder. The b is bladder and the u is ureter. (**D**–**F**) Visible light image, near-infrared fluorescence image, and fusion image of the intestine. The i is intestine.

1**.** In the simultaneous illumination of the surgical area with visible light and ICG excitation light, the naked eye can only perceive the tissue's appearance under visible light. Meanwhile, the visible light camera captures the natural light image of the tissue, and the near-infrared camera records the fluorescent image of the ICG-labeled tissue. These two images seamlessly merge in real-time on LabVIEW software. Consequently, the surgeon gains a clear view of the ICG-labeled tissue imaging through the computer screen. Figure 5 showcases the fluorescence imaging of the ureter (Fig. 5Au), bladder (Fig. 5Ab), and intestine (Fig. 5Di), respectively. Given that urinary system organs are predominantly situated in the retroperitoneum with deep anatomical positions, the Indocyanine Green Video Angiography (ICGVA) technology proves instrumental in mitigating or preventing side damage to the ureter and bladder during abdominal surgery.

**Figure 6:**
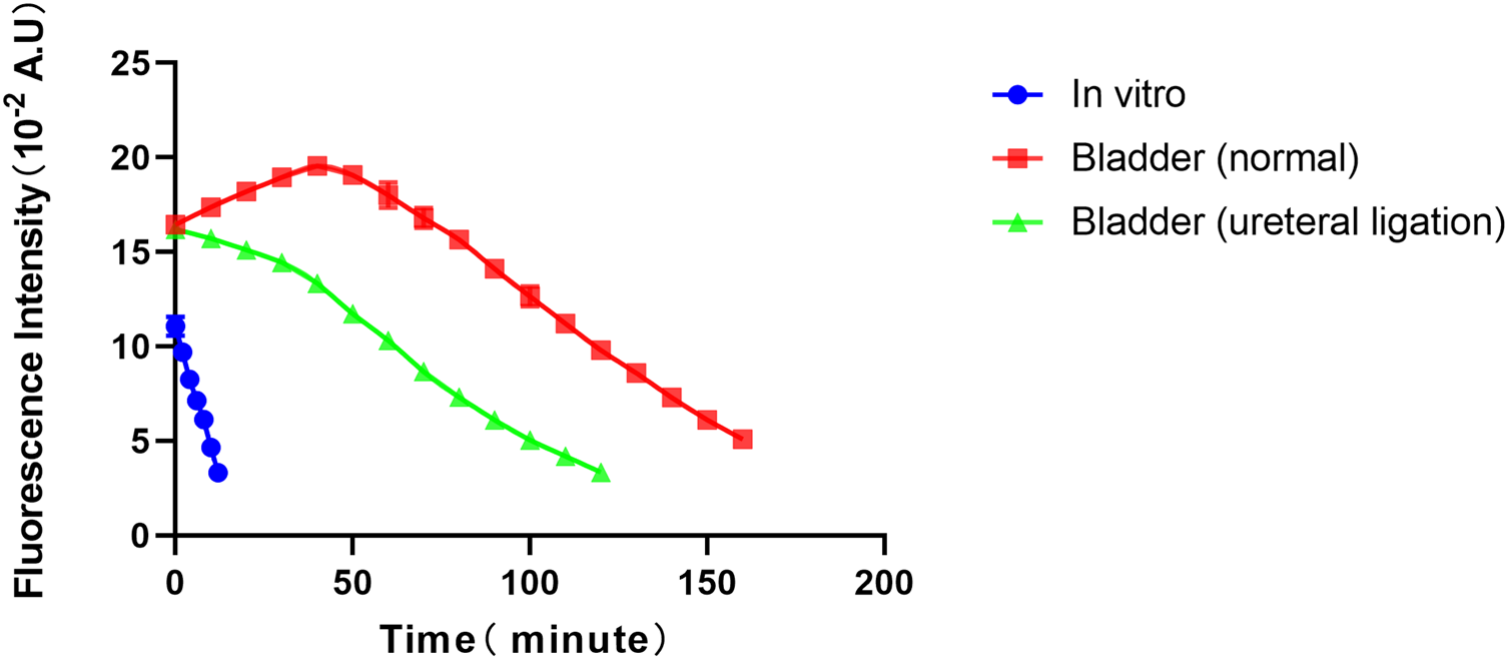
The three curves illustrate the average fluorescence intensity of 5 ml 0.01 mg/ml ICG in vitro, bladder (normal), and bladder (ureteral ligation) over time.

2. In vitro, 5 ml of 0.01 mg/ml ICG exhibits a notable decrease in fluorescence intensity, becoming faint after 12 min. Upon performing bilateral ureteral ligation, the initial fluorescence intensity of 5 ml 0.01 mg/ml ICG in the bladder surpasses that outside the bladder, with a gradual decline in fluorescence intensity. By the 120-min mark, the bladder's brightness ceases to offer assistance for surgery. Conversely, when the ureter remains unobstructed, the average fluorescence intensity of 5 ml 0.01 mg/ml ICG in the bladder exhibits an initial increase followed by a subsequent decrease. The peak fluorescence intensity occurs at 40 min, remains constant at 70 min, and by 130 min, the bladder's brightness is no longer conducive to surgical guidance (Fig. 6).

## Discussion

The near-infrared fluorescence imaging equipment, utilizing Indocyanine Green (ICG), has seen widespread use in surgical procedures. However, the associated equipment^13^ presents challenges with high application requirements, elevated equipment costs, and limited mobility. This equipment represents a reconstruction and enhancement of the near-infrared fluorescence imaging equipment initially designed by Grand et al.^12^. We have implemented an adjustable focus vision platform to facilitate swift and precise image acquisition in both experimental settings and surgical procedures. Opting for LED light sources over halogen alternatives, we benefit from increased brightness, reduced heat generation, prolonged service life, and simplified control. The inclusion of air-cooled radiators further ensures luminous stability. Additionally, we have chosen a V-interface line scanning lens and the BIGEYE10000KPA camera to enhance adaptability and compatibility. The accessories needed for the entire system can be flexibly adjusted to meet specific requirements. The overall equipment design is straightforward, easily detachable, and boasts excellent mobility. Looking ahead, its application extends beyond operating rooms, encompassing diverse scenarios and environments, including those where outdoor conditions are lacking in traditional surgical settings.

Our device has undergone pertinent animal experiments, successfully accomplishing fluorescence imaging of the rabbit's bladder, ureter, and small intestine, thereby validating the imaging functionality of the device (refer to Fig. 5). The experimental outcomes, as depicted in Fig. 6, affirm that biological structures, such as the bladder, can influence the fluorescence intensity variations of Indocyanine Green (ICG). In its aqueous solution, ICG exhibits a low level of fluorescence, but upon binding to hydrophobic pockets of proteins (such as albumin) or cellular membranes, its emission increases^14^. In comparison to in vitro conditions, Indocyanine Green (ICG) not only elevates the initial fluorescence intensity in the bladder but also extends the duration of fluorescence imaging. Furthermore, urine presence introduces a discernible impact on fluorescence imaging. When both ureters remain unobstructed, the fluorescence intensity of 0.01 mg/ml ICG in the bladder exhibits a pattern of initial increase followed by a subsequent decrease. The bladder's fluorescence intensity can persist above the initial level for 70 min, offering valuable insights for the application of ICG in bladder-related surgeries. For instance, in cystectomy for bladder cancer surgery, retrograde injection of ICG aids in determining the position and complete contour of the bladder during surgery, facilitating complete or partial cystectomy and minimizing the risk of tumor implantation^15^. Beyond the 70-min mark in surgery duration, a reinjection of ICG becomes necessary to sustain a heightened level of fluorescence intensity in the bladder.

The present equipment still exhibits certain drawbacks and limitations. Complete procurement of equipment components is not feasible in a single transaction, and parameters require manual adjustment. There is a need to enhance the frame rate. Currently, the device is exclusively employed in animal experiments and has not yet undergone utilization in clinical surgery to establish its feasibility. Additionally, the device is constructed within the framework of the first near-infrared wavelength window (NIR-I, wavelength 700–900 nm). Ongoing research indicates that, when compared to NIR-I, the second near-infrared wavelength window (NIR-II, 1000–1700 nm) can heighten contrast in fluorescence imaging at millimeter depth with micrometer resolution^16^. For instance, in liver tumor surgery, NIR-II offers increased tumor detection sensitivity, a higher tumor-to-normal liver tissue signal ratio, and an elevated tumor detection rate. In the future, devices based on NIR-II enhancements may hold more guiding significance for various clinical surgeries.

## Data Availability

All data generated or analysed during this study are included in this published article [and its supplementary information files].
